# Perioperative Outcomes of Lung Cancer Surgery in Women: A Canadian Nationwide Retrospective Cohort Study

**DOI:** 10.1093/ejcts/ezaf236

**Published:** 2025-07-12

**Authors:** Holly T Philpott, Rowan Murphy, Cassidy McCausland, Yingtong Gao, Caitlin Anstee, Molly Gingrich, Andrew Seely, Alison Wallace, The CATS Database Working Group, Andrew Seely, Christian Finley, Tom Waddell, Lorenzo Ferri, Caitlin Anstee, Molly Gingrich

**Affiliations:** Department of Surgery, Division of Thoracic Surgery, Dalhousie University, Halifax, NS, B3H 2Y9, Canada; Department of Surgery, Division of Thoracic Surgery, Dalhousie University, Halifax, NS, B3H 2Y9, Canada; Department of Surgery, Division of Thoracic Surgery, Dalhousie University, Halifax, NS, B3H 2Y9, Canada; Department of Surgery, Division of Thoracic Surgery, Dalhousie University, Halifax, NS, B3H 2Y9, Canada; Department of Surgery, Division of Thoracic Surgery, University of Ottawa, The Ottawa Hospital, Ottawa, ON, K1H 8L6, Canada; Department of Surgery, Division of Thoracic Surgery, University of Ottawa, The Ottawa Hospital, Ottawa, ON, K1H 8L6, Canada; Department of Surgery, Division of Thoracic Surgery, University of Ottawa, The Ottawa Hospital, Ottawa, ON, K1H 8L6, Canada; Department of Critical Care Medicine, The Ottawa Hospital, Ottawa, ON, K1Y 4E9, Canada; Department of Surgery, Division of Thoracic Surgery, Dalhousie University, Halifax, NS, B3H 2Y9, Canada; Department of Pathology, Dalhousie University, Halifax, NS, B3H 4R2, Canada

**Keywords:** lung cancer surgery, sex differences, perioperative outcomes, postoperative complications

## Abstract

**Objectives:**

Sex differences in perioperative outcomes following lung cancer surgery remain understudied. This study evaluated these differences in a national cohort.

**Methods:**

Data for patients who underwent lung cancer surgery between January 2017-December 2022 at 13 hospitals were extracted from the Canadian Association of Thoracic Surgeons National Database. Preoperative characteristics, surgery-related, tumour-related, and postoperative outcomes data were collected. Mixed-effects logistic regression models were used to determine perioperative variables associated with female sex.

**Results:**

A total of 9922 patients were included, and 55.4% were female. Female patients had higher rates of minor complications, lower rates of major complications, and lower mortality. Females were less likely to be active smokers (odds ratio [OR] = 0.66; 95% confidence interval [CI], 0.52, 0.83), have comorbidities, have squamous cell carcinoma (OR = 0.34; 95% CI, 0.26, 0.44), or an air leak complication postoperatively (OR = 0.66; 95% CI, 0.51, 0.86). However, females with chronic obstructive pulmonary disease (COPD) or squamous cell carcinoma had higher odds of experiencing an air leak complication postoperatively.

**Conclusions:**

Females had fewer comorbidities, less advanced stage cancer, less pulmonary resection, and different tumour types, all leading to lower rates of major complications and mortality compared to males. Understanding preoperative factors that contribute to sex differences in adverse events can enhance the short- and long-term outcomes for patients with lung cancer.

## Graphical Abstract

**Figure:**
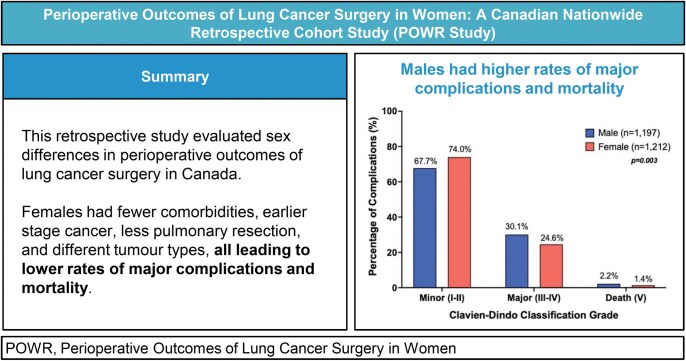

## INTRODUCTION

Lung cancer is highly lethal and in Canada is responsible for 1 in 4 cancer-related deaths,^1^^,^^2^ contributing to more deaths than breast, colon, and prostate cancer combined.^1^^,^^2^ The epidemiology of lung cancer is changing in terms of sex differences in incidence. In recent decades, lung cancer incidence rates have decreased or plateaued in males but have risen among females.^3–6^

Although sex differences in treatment outcomes and survival have been studied in lung cancer, few studies have investigated sex differences in perioperative outcomes. Since surgery is the primary treatment for early-stage lung cancer, uncovering potential sex differences in perioperative outcomes will help to improve patient-specific management. A recent study by Fibla *et al.*^7^ showed the number and severity of postoperative complications were increased in males compared to females in a Spanish population; however, this type of study has not been completed in Canada. Importantly, adverse events are a major cause of harm for patients undergoing an already high-stakes surgery. Adverse events increase length of stay, worsen patient experience, decrease quality of life, and cost the healthcare system tens of thousands of dollars. Moreover, females are more likely to undergo surgical resection compared to men,^8^ further highlighting the importance of studying sex differences in perioperative outcomes in lung cancer surgery.

With the goal of improving patient-specific management of lung cancer and quality of life according to sex, the purpose of this study is to assess sex differences in the perioperative profile for patients undergoing lung cancer surgery in Canada.

## METHODS

### Study design, data sources, and study sample

This is a Canadian multicentre retrospective cohort study of patients who have undergone lung cancer surgery. The study protocol was approved by the Ottawa Health Science Network Research Ethics Board (Protocol ID 20230057-01H). Patient consent was waived to facilitate creation of national database and access to the database is limited and password protected.

Data were extracted from the Canadian Association of Thoracic Surgeons (CATS) database, a national thoracic surgery database established in 2017 (see https://ottawatmm.org/), for all patients who underwent lung cancer surgery from January 2017-December 2022. Thirteen centres across Canada (11 teaching hospitals and 2 community hospitals) were included out of a total of 30 accredited thoracic surgery centres representing all provinces in Canada. Patients undergoing exploratory, palliative, or emergency life-saving surgeries and pulmonary metastasectomies were excluded. Quality control processes are in place at local sites and centrally for the CATS database to ensure data accuracy.

Data for all desired outcomes were not consistently captured across all centres. As a result, the number of patients included in each analysis are reported separately to reflect the available data for each outcome.

### Outcome measures

Preoperative general characteristics included sex, age, body mass index (BMI), smoking status (active, former, and never), and functional status (independent, partially dependent, and totally dependent). Comorbidities included obesity (BMI > 30 kg/m^2^), chronic obstructive pulmonary disease (COPD), diabetes, hypertension, coronary artery disease, congestive heart failure, and peripheral vascular disease. Pulmonary function, Forced Expiratory Volume in 1-second (FEV1%) and Diffusion Capacity of the Lungs (DLCO%), were also included.

Surgical data included resection type and intraoperative complications. Tumour-related variables included TNM stage (2017 TNM Classification, 8th edition)^9^ and histopathological morphology.

Postoperative data included length of stay and postoperative complications. The severity of complications were graded according to Clavien-Dindo classification.^10^^,^^11^

### Statistical analyses

Patients were stratified by sex. The Student’s t-test was used to compare continuous variables and Chi square test was used to compare categorical variables between sexes. Results of t-tests were reported as mean difference with 95% confidence intervals (CIs), and results of Chi square test were reported as *P* value, which were 2-tailed and not adjusted for multiplicity.

We fitted a series of mixed-effects logistic regression models to test the association of potential perioperative predictors with sex. We performed analyses using complete-case data, which primarily were from hospital sites including Vancouver, UHN, Ottawa, and Nova Scotia. We also completed analyses where missing data were addressed with multiple imputation by chained equations (MICE). Cases with < 50% missing data were retained in the dataset. Twenty imputed datasets were created and analysed using Rubin’s rules to combine estimates. Predictors of interest included age, smoking status, comorbidities, tumour histology, tumour pathological stage, and air leak complication (yes/no). All models were adjusted for hospital site (random effect). Results were reported as odds ratios (ORs) with 95% CIs. We performed a sensitivity analysis on the complete-case dataset using propensity score matching to assess robustness of association between sex and prolonged air leak complication.

In secondary analyses, we investigated sex differences in the association of perioperative predictors with prolonged air leak. We fitted a series of mixed-effects logistic regression models, adjusting for hospital site (random effect). Analyses were performed using both complete-case and MICE as described above. Results were reported as ORs with 95% CIs.

Analyses were completed using StataIC 15.1 (StatCorp) software. A *P* value of < 0.050 indicated statistical significance and 95% CIs as a measure of precision.

### Data availability

The data will be shared on reasonable request to the corresponding author.

## RESULTS

A total of 5499 females and 4423 males were included. No statistically significant differences in age, BMI, or functional status between males and females were identified. However, a higher proportion of females were non-smokers (**Table 1**). Male participants had higher rates of almost all measured comorbidities, except obesity. Female participants demonstrated preserved pulmonary function compared to male participants (**Table 1**).

**Table 1.** Preoperative Clinical Characteristics of Patients According to Sex

Variables	Total	Male	Female	*P* value
	(*n* = 9922)	(*n *= 4423)	(*n* = 5499)	
Age, years, mean ± SD	66.6 ± 10.9 (18-95)	66.8 ± 11.7 (18-95)	66.5 ± 10.2 (18-95)	0.28 ± 0.22 (−0.15, 0.71) *P *= 0.207
Smoking status, *n* (%)	Total	Male	Female	
	(*n* = 6221)	(*n* = 2771)	(*n* = 3448)	
Never	1600 (25.7%)	557 (20.1%)	1043 (30.3%)	*P* ** < 0.001**
Active	1264 (20.3%)	608 (21.9%)	656 (19.0%)	
Former	3357 (54.0%)	1606 (58.0%)	1749 (50.7%)	
	Total	Male	Female	
	(*n* = 4674)	(*n* = 2028)	(*n* = 2646)	
BMI, kg/m^2^, mean ± SD	26.8 ± 5.8 (12.7-69.6)	27.0 ± 5.5 (15-69.4)	26.7 ± 6.1 (12.7-69.6)	0.29 ± 0.17 (−0.05, 0.63) *P* = 0.091
Functional status, *n* (%)	Total	Male	Female	
	(*n* = 5581)	(*n* = 2544)	(*n* = 3037)	
Independent	5351 (95.9%)	2441 (96.0%)	2910 (95.8%)	*P *= 0.968
Partially dependent	219 (3.9%)	98 (3.8%)	121 (4.0%)	
Totally dependent	11 (0.2%)	5 (0.2%)	6 (0.2%)	
Comorbidities, *n* (%)	Total	Male	Female	
Obesity, BMI >30 kg/m^2^	1193 (25.5%) *n* = 4674	500 (24.7%) *n* = 2028	693 (26.2%) *n* = 2646	*P *= 0.233
COPD	2093 (25.0%) *n* = 8376	1007 (25.7%) *n* = 3926	1086 (24.4%) *n* = 4450	*P *= 0.189
Diabetes	1580 (17.6%) *n* = 9003	843 (19.9%) *n* = 4195	746 (15.5%) *n* = 4808	*P* ** < 0.001**
Hypertension	4218 (48.5%) *n* = 8698	2131 (52.1%) *n* = 4087	2087 (45.3%) *n* = 4611	*P* ** < 0.001**
Coronary artery disease	809 (17.8%) *n* = 4534	494 (25.1%) *n* = 1970	315 (12.3%) *n* = 2564	*P* ** < 0.001**
Congestive heart failure	64 (1.6%) *n* = 4061	41 (2.4%) *n* = 1737	23 (1.0%) *n* = 2324	*P* ** < 0.001**
Peripheral vascular disease	185 (4.6%) *n* = 4069	117 (6.7%) *n* = 1745	68 (2.9%) *n* = 2324	*P* ** < 0.001**
Lung function tests, mean ± SD (range)	Total	Male	Female	
	(*n* = 3453)	(*n* = 1500)	(*n *= 1953)	
FEV1%	83.1 ± 18.5 (12-153)	81.4 ± 17.8 (28-139)	84.4 ± 18.9 (12-153)	−2.97 ± 0.63 (−4.21, −1.73) *P* **< 0.001**
	Total	Male	Female	
	(*n* = 3304)	(*n* = 1436)	(*n* = 1868)	
DLCO%	78.6 ± 19.0 (12-166)	77.6 ± 18.5 (19-159)	79.3 ± 19.4 (12-166)	−1.71 ± 0.67 (−3.02, −0.40) *P* **= 0.010**

Continuous data were compared using Student’s t-test and reported as mean difference (95% CIs) and *P* value. Categorical data were compared using a Chi square test and reported as *P* value. Bolded indicates statistical significance. Abbreviation: BMI: body mass index; DLCO%: Diffusion Capacity of the Lungs; FEV1%: Forced Expiratory Volume in 1-second.

Female patients were more likely to undergo a lobectomy and less likely to undergo pneumonectomy compared to male patients (*P* < 0.001). There was a total of 195 intraoperative complications reported. Males had higher rates of intraoperative bleeding and cardiac arrest compared to female patients (**Table 2**).

**Table 2.** Surgical-related Variables According to Sex

Resection type, *n* (%)	Total cohort	Male	Female	
	(*n* = 8598)	(*n *= 3818)	(*n* = 4780)	
Lobectomy	5377 (62.5%)	2273 (59.5%)	3104 (64.9%)	*P* ** < 0.001**
Wedge resection	1912 (22.2%)	896 (23.5%)	1016 (21.3%)	
Segmentectomy	646 (7.5%)	277 (7.3%)	369 (7.7%)	
Extended lobectomy	298 (3.5%)	163 (4.3%)	135 (2.8%)	
Bilobectomy	92 (1.1%)	44 (1.2%)	48 (1.0%)	
Pneumonectomy	273 (3.2%)	165 (4.3%)	108 (2.3%)	

Intraoperative complications, *n* (%)	Total cohort	Male	Female	
	*n* = 196	*n* = 109	*n* = 87	
Arrhythmia	12 (6.1%)	6 (50.0%)	6 (50.0%)	*P *= 0.719
Bleeding	166 (84.5%)	92 (55.4%)	74 (44.5%)	***P *= 0.006**
Cardiac arrest	4 (2.0%)	4 (100.0%)	0 (0.0%)	***P *= 0.026**
Injury to airway	14 (7.1%)	7 (50.0%)	7 (50.0%)	*P *= 0.664

Intraoperative complication percentages are of column totals for total cohort. By sex intraoperative complication percentages are of row totals. Continuous data were compared using Student’s t-test and reported as mean difference (95% CIs) and *P* value. Categorical data were compared using a Chi square test and reported as *P* value. Bolded indicates statistical significance.

Male patients had decreased frequency of adenocarcinoma and carcinoid histopathological types and increased frequency of squamous cell carcinoma compared to females (*P* < 0.001) (**Table 3**). Female patients had higher rates of T1 stage tumours. Male patients had higher rates of spread to regional lymph nodes (N1 or N2) than female patients (**Table 3**).

**Table 3.** Tumour-related Characteristics by Sex

Histological type, *n* (%)	Total cohort	Male	Female	*P* value
	(*n* = 4556)	(*n* = 1931)	(*n* = 2625)	
Adenocarcinoma	3352 (73.6%)	1347 (69.8%)	2005 (76.4%)	*P* ** < 0.001**
Squamous cell carcinoma	694 (15.2%)	391 (20.3%)	303 (11.5%)	
Carcinoid	297 (6.5%)	96 (5.0%)	201 (7.7%)	
Other	213 (4.7%)	97 (5.0%)	116 (4.4%)	

T (tumour size), *n* (%)	Total cohort	Male	Female	
	(*n* = 5131)	(*n* = 2199)	(*n* = 2932)	
T1mis/Tis	170 (3.3%)	53 (2.4%)	117 (4.0%)	*P* ** < 0.001**
T1A	969 (18.9%)	390 (17.7%)	579 (19.8%)	
T1B	1427 (27.8%)	558 (25.4%)	869 (29.6%)	
T1C	368 (7.2%)	167 (7.6%)	201 (6.9%)	
T2A	1215 (23.7%)	515 (23.4%)	700 (23.8%)	
T2B	285 (5.6%)	148 (6.7%)	137 (4.7%)	
T3	487 (9.5%)	249 (11.3%)	238 (8.1%)	
T4	210 (4.1%)	119 (5.4%)	91 (3.1%)	

*N* (regional lymph nodes), *n* (%)	Total cohort	Male	Female	
	(*n* = 4526)	(*n* = 1967)	(*n* = 2559)	
N0	3659 (80.8%)	1554 (79.0%)	2105 (82.3%)	***P *= 0.010**
N1	561 (12.4%)	276 (14.0%)	285 (11.1%)	
N2	306 (6.8%)	137 (7.0%)	169 (6.6%)	

Continuous data were compared using Student’s t-test and reported as mean difference (95% CIs) and *P* value. Categorical data were compared using a Chi square test and reported as *P* value. Bolded indicates statistical significance. Discrepancies in *n* values are due to availability of pathology reports.

The average length of hospital stay was 0.70 days longer in male patients compared to female patients (*P* < 0.001) (**Table 4**). Male patients had higher rates of pneumonia, respiratory distress/failure, bronchopleural fistula, effusion, empyema, and arrhythmia compared to female patients; however, these differences were not statistically significant (*P *= 0.059) (**Table 4**).

**Table 4.** Postoperative Complications of Lung Cancer Surgery by Sex

Postoperative complication, *n* (%)	Total number	Male	Female	*P* value
	*n* = 2262	*n* = 1179	*n* = 1082	
Prolonged alveolar air leak	1030 (45.5%)	510 (43.3%)	520 (48.1%)	*P *= 0.059
Pneumonia	268 (11.9%)	142 (12.0%)	125 (11.6%)	
Atelectasis	72 (3.2%)	31 (2.6%)	41 (3.8%)	
Respiratory distress/failure	135 (6.0%)	83 (7.0%)	52 (4.8%)	
Bronchopleural fistula	25 (1.1%)	17 (1.4%)	8 (0.7%)	
Effusion	116 (5.1%)	64 (5.4%)	52 (4.8%)	
Empyema	88 (3.9%)	54 (4.6%)	34 (3.1%)	
Hemoptysis	7 (0.3%)	3 (0.3%)	4 (0.4%)	
Arrhythmia	478 (21.1%)	252 (21.4%)	226 (20.9%)	
Infection	43 (1.9%)	23 (2.0%)	20 (1.9%)	
Length of hospital stay, days, mean ± SD (range)	5.0 ± 8.0 (0.0-268.0) *n* = 9062	5.4 ± 8.8 (0.0-268.0) *n* = 4044	4.7 ± 7.2 (0.0-205.0) *n* = 5018	0.70 (0.38, 1.03) *P* **< 0.001**

Postoperative complication percentages are of column totals. Continuous data were compared using Student’s t-test and reported as mean difference (95% CIs) and *P* value. Categorical data were compared using a Chi square test and reported as *P* value. Bolded indicates statistical significance.

**Figure 1.**
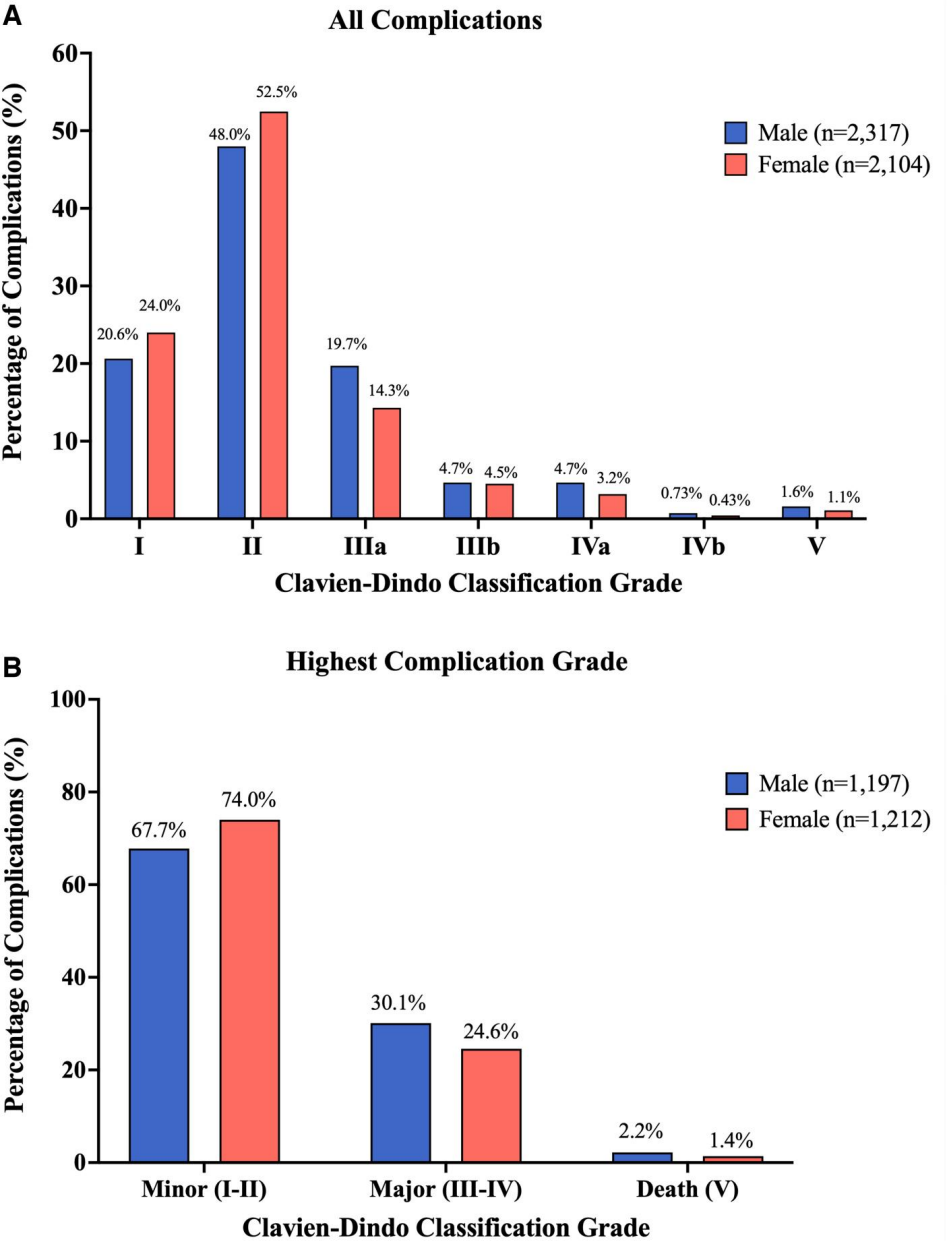
Frequency Distributions of Postoperative Complications According to Severity (Clavien-Dindo Classification) in Males and Females with Lung Cancer. (A) All complication grades in cohort separated by sex. (B) Highest complication grade for each patient separated by sex, to account for patients with more than 1 postoperative complication.

When comparing all complications, females had higher rates of Clavien-Dindo Grade I and II and lower rates of Clavien-Dindo Grade III and above (*P* < 0.001; **Figure 1A**). To account for patients with more than 1 postoperative complication, we compared highest complication grade. Similarly, females had higher rates of minor (grade I and II) and lower rates of major (Grade III-IV) complications and death (*P* = 0.003; **Figure 1B**).

Active smoking had lower odds of being associated with female sex compared to male sex (OR: 0.66; 95% CI, 0.52, 0.83). In addition, comorbidities such as diabetes, COPD, coronary artery disease, and peripheral vascular disease were associated with lower odds of female sex. Squamous cell carcinoma was associated with lower odds of female sex (OR: 0.34; 95% CI, 0.26, 0.44). T1A tumour stage was associated with higher odds of female sex (OR: 1.28; 95% CI, 0.98, 1.68); however, not statistically significant. Female sex was associated with lower odds of having a postoperative air leak complication. The results obtained from multiple imputation were largely consistent with those from complete-case analysis, suggesting missing data did not meaningfully bias the results (**Table 5**).

**Table 5.** Series of Mixed-effects Logistic Regression Models

Model	Complete case odds ratio	Multiple imputation odds ratio
Age	0.99 (0.98-1.00)	1.00 (0.99-1.00)
Active smoker	**0.66 (0.52**-**0.83)**	**0.77 (0.65**-**0.92)**
Diabetes	**0.69 (0.54**-**0.87)**	**0.70 (0.59**-**0.84)**
COPD	**0.74 (0.59**-**0.91)**	**0.82 (0.70**-**0.96)**
Coronary artery disease	**0.40 (0.29**-**0.55)**	**0.40 (0.32**-**0.50)**
Peripheral vascular disease	**0.44 (0.27**-**0.71)**	**0.39 (0.26**-**0.57)**
Squamous cell carcinoma	**0.34 (0.26**-**0.44)**	**0.43 (0.34**-**0.53)**
T1A stage pathology	1.28 (0.98-1.68)	**1.31 (1.06**-**1.63)**
Air leak	**0.66 (0.51**-**0.86)**	**0.69 (0.55**-**0.85)**

Comparison from complete case (male = 726; female = 1113) and multiple imputation (male = 1430; female = 1976) analyses. Adjusting for variance by hospital site (random effect). Multiple imputation by chained equations (MICE) was used to address missing values. Bolded indicates statistical significance. Abbreviation: COPD: chronic obstructive pulmonary disease.

We performed a sensitivity analysis using propensity score matching. Female and male patients were matched 1:1 based on age, smoking status, comorbidities, tumour stage, and tumour histology. After matching, the ATT estimate was −6.7% (SE = 0.036, t = −1.86), indicating a lower incidence of air leak among females. Although this result was not statistically significant, the trend is consistent with the primary findings. In addition, we performed a weighted logistic regression on the propensity score-matched sample. Female sex was associated with significantly lower odds of an air leak complication (OR: 0.61; 95% CI, 0.48, 0.76).

Female patients with COPD or squamous cell carcinoma had higher odds of experiencing an air leak complication postoperatively (**Table 6**). Males with COPD had higher odds of experiencing an air leak complication. In addition, male patients who were active smokers were more likely to experience air leak complication; however, this was not statistically significant. Male patients with diabetes were less likely to experience an air leak complication. The results obtained from multiple imputation were consistent with the complete-case analysis, suggesting missing data did not meaningfully bias the results (**Table 6**).

**Table 6.** Sex-based Analysis Using a Series of Mixed-effects Logistic Regression Models

Female (*n* = 1113 complete case; *n* = 1976 multiple imputation)		
Model	**Complete case Odds ratio** (95% CI)	**Multiple imputation Odds ratio** (95% CI)
Age	1.02 (0.99-1.04)	1.03 (1.01-1.05)
Active smoker	0.98 (0.63-1.53)	0.72 (0.44-1.19)
Diabetes	0.82 (0.51-1.36)	0.63 (0.36-1.10)
**COPD**	**1.96 (1.36**-**2.82)**	**2.29 (1.91**-**2.75)**
Coronary artery disease	0.54 (0.21-1.37)	0.47 (0.20-1.11)
Peripheral vascular disease	0.46 (0.11-1.95)	0.63 (0.13-2.98)
**Squamous cell carcinoma**	**2.07 (1.27**-**3.37)**	**1.83 (1.06**-**3.17)**
T1A stage pathology	0.78 (0.47-1.31)	0.74 (0.46-1.19)

Male (*n* = 726 complete case; *n* = 1430 multiple imputation)		
Model	Complete case Odds ratio (95% CI)	Multiple imputation Odds ratio (95%CI)
Age	1.01 (0.99-1.04)	1.01 (0.99-1.03)
Active smoker	1.49 (0.99-2.23)	1.43 (1.00-2.03)
**Diabetes**	**0.58 (0.34**-**0.96)**	**0.59 (0.39**-**0.90)**
**COPD**	**2.01 (1.37**-**2.96)**	**1.68 (1.21**-**2.33)**
Coronary artery disease	1.18 (0.69-2.02)	0.92 (0.60-1.40)
Peripheral vascular disease	0.90 (0.39-2.08)	0.72 (0.31-1.67)
Squamous cell carcinoma	0.99 (0.64-1.54)	1.02 (0.67-1.56)
T1A stage pathology	0.97 (0.56-1.71)	0.92 (0.57-1.48)

Adjusting for variance by hospital site (random effect). Multiple imputation by chained equations (MICE) was used to address missing values. Bolded indicates statistical significance. Abbreviation: COPD: chronic obstructive pulmonary disease.

## DISCUSSION

This nationwide multicentre retrospective cohort study highlighted sex differences in perioperative profile. Female patients had decreased smoking history, lower rates of comorbidities, and increased preoperative lung function. Further, we identified sex differences in tumour histology and postoperative complication rates.

Our findings suggest that female patients were more likely to be non-smokers. This finding aligns with recent findings from a Spanish cohort^7^ and older studies.^12^^,^^13^ Females may have increased susceptibility to the adverse effects of carcinogens found in tobacco,^14^ making even small amounts of tobacco exposure significant to their lung cancer outcomes; however, there are conflicting results on this phenomenon.^14^

We found sex differences in tumour histology, where males had almost twice the rate of squamous cell carcinoma. Similar to previous literature,^14–16^ the commonest tumour in females was adenocarcinoma. Although we were not able to assess tumour mutations, an *EGFR* gene mutation has been associated with adenocarcinoma in females.^17–19^ Some literature suggests sex differences in tumour histopathology may be related to hormones, sex-specific metabolism of carcinogens, and efficiency of DNA damage repair mechanisms^20^; however, it is not entirely clear what is driving sex differences in tumour histopathology and how this influences prognosis and should be explored further.

Herein, females had higher rates of minor complications, lower rates of major complications and mortality, and shorter length of stay. This is concordant with previous literature that found differences in length of stay, 30- and 90-day mortality, and re-admission rates.^7^^,^^21^ Evidence suggests that postoperative complications may be related to age, squamous cell carcinoma, and incidence of comorbidities.^7^^,^^22^ Similarly, we found that female sex was associated with lower odds of comorbidities, postoperative air leak complication, and squamous cell carcinoma. Female patients trended towards higher frequency of T1A tumour stage (early). These observations raise the hypothesis that earlier diagnosis and/or lower exposure to smoking in females may contribute to lower comorbidity burden and, consequently, differences in postoperative outcomes. Although females were less likely to be active smokers and less likely to have squamous cell carcinoma, we found that females with COPD and females those with squamous cell carcinoma had higher odds of an air leak complication, and these associations differed from males. This suggests that certain comorbidities, health-related habits, and tumour histology may differentially influence course of disease and recovery based on sex. Additionally, male patients with diabetes were less likely to have an air leak complication. We speculate that patients with diabetes demonstrated preserved lung function, likely because those with significant comorbidities were considered high-risk and thus excluded from surgical intervention. This selection bias may have resulted in a cohort of diabetic patients with better baseline health, including lung function, compared to those without diabetes.

Although we were not able to measure short- or long-term survival after surgery, cohort studies from other countries have shown that there are differences in survival postoperatively in females.^8^^,^^23–25^ In a Finnish study, survival advantage was more apparent in the early postoperative period but remained increased at 5- and 10-year follow up.^8^ One study suggested that the difference in survival may be due to age, where they found female patients tended to present younger^25^; however, this was not the case in our cohort, as there was no difference in age. Interestingly, a Norwegian study found that female sex was associated with improved prognosis independent of age, tumour stage or histology, and operation type.^26^ Other studies have also shown there is a prognostic benefit of female sex not only in early-stage disease but at all stages of disease.^27^^,^^28^ Although there are higher resection rates in female patients, males had significantly decreased survival.^8^ Multiple studies suggest that males may be poorer surgical candidates compared to females given smoking status, lung function, and comorbidity profiles.^7^^,^^8^^,^^29^^,^^30^ This may partially explain why females in our cohort had higher rates of minor complications but lower rates of major complications. Whereas male patients are at increased risk of poorer outcomes postoperatively and decreased long-term survival. There is a need to optimize health of individuals, specifically targeting males, seeking surgery for lung cancer in the preoperative period to promote better outcomes overall. Prehabilitation programs offer a promising strategy to enhance surgical readiness through targeted interventions such as physical activity, nutrition, and support for smoking cessation.^31^

There are several limitations to this study. The proportion of patients captured in the database has increased over time due to the onboarding of additional thoracic surgery centres after 2017, which may introduce temporal biases. In addition, data completeness varied across hospital sites, with some centres contributing only core perioperative data and others providing more detailed information. As a result, the sample size varied between analyses depending on availability of specific outcome measures. This heterogeneity in data capture may limit the consistency of some outcome measures. Finally, the missing data appear to be missing at random and driven by site-level differences in data collection practices rather than patient characteristics. Although we accounted for this by including all complete data in our analyses, bias from missing data cannot be excluded. Our sample consisted of major thoracic surgery centres across Canada, which improves the generalizability of the study findings; however, the results should be interpreted with the above limitations in mind.

In conclusion, female patients have better preoperative comorbidity profiles, present with lower grade tumours, and have fewer major complications and lower mortality rates postoperatively. These findings align with similar work done in other countries. By understanding the perioperative factors that contribute to sex differences in adverse events, we will be able to create better risk management plans for patients with lung cancer, with the goal of improving quality of life and survival.

## Data Availability

The data will be shared on reasonable request to the corresponding author.

## The CATS Database Working Group

Andrew Seely, Christian Finley, Tom Waddell, and Lorenzo Ferri—Project Co-leads—CATS Database and Quality Improvement Program; Caitlin Anstee—Database/Software Manager—TSDMC; and Molly Gingrich—Research Assistant—TSDMC.

## References

[1] Brenner DR , Poirier A, Woods RR, et al; Canadian Cancer Statistics Advisory Committee. Projected estimates of cancer in Canada in 2022. CMAJ. 2022;194:E601-E607. 35500919 10.1503/cmaj.212097 PMC9067380

[2] Canadian Cancer Statistics Advisory Committee. Canadian Cancer Statistics 2019. Toronto, ON: Canadian Cancer Society; 2019. Accessed November 2024. Available at: cancer.ca/Canadian-Cancer-Statistics-2019-EN

[3] Torre LA , Siegel RL, Jemal A. Lung cancer and personalized medicine, current knowledge and therapies. Adv Exp Med Biol. 2015;893:1-19. 10.1007/978-3-319-24223-1_1 26667336

[4] Matteis SD , Consonni D, Pesatori AC, et al Are women who smoke at higher risk for lung cancer than men who smoke? Am J Epidemiol. 2013;177:601-612. 23425629 10.1093/aje/kws445 PMC3657535

[5] Remon J , Molina-Montes E, Majem M, et al Lung cancer in women: an overview with special focus on Spanish women. Clin Transl Oncol. 2014;16:517-528. 24277573 10.1007/s12094-013-1137-7

[6] Janssen-Heijnen MLG , Coebergh J-WW. The changing epidemiology of lung cancer in Europe. Lung Cancer. 2003;41:245-258. 12928116 10.1016/s0169-5002(03)00230-7

[7] Fibla JJ , Molins L, Quero F, et al Perioperative outcome of lung cancer surgery in women: results from a Spanish nationwide prospective cohort study. J Thorac Dis. 2019;11:1475-1484. 31179090 10.21037/jtd.2019.03.30 PMC6531693

[8] Lautamäki A , Gunn J, Sipilä J, et al Women have a higher resection rate for lung cancer and improved survival after surgery. Interact CardioVasc Thorac Surg. 2021;32:889-895. 33523210 10.1093/icvts/ivab006 PMC8923395

[9] Detterbeck FC , Boffa DJ, Kim AW, et al The Eighth edition lung cancer stage classification. Chest. 2017;151:193-203. 27780786 10.1016/j.chest.2016.10.010

[10] Dindo D , Demartines N, Clavien P-A. Classification of surgical complications. Ann Surg. 2004;240:205-213. 15273542 10.1097/01.sla.0000133083.54934.ae PMC1360123

[11] Seely AJE , Ivanovic J, Threader J, et al Systematic classification of morbidity and mortality after thoracic surgery. Ann Thorac Surg. 2010;90:936-942; discussion 942. 20732521 10.1016/j.athoracsur.2010.05.014

[12] Toh C-K , Gao F, Lim W-T, et al Never-smokers with lung cancer: epidemiologic evidence of a distinct disease entity. J Clin Oncol. 2006;24:2245-2251. 16710022 10.1200/JCO.2005.04.8033

[13] Wheatley-Price P , Blackhall F, Thatcher N. The influence of sex in non-small cell lung cancer. Oncol Res Treat. 2009;32:547-548. 10.1159/000235609 19816068

[14] Barrera R , Fuentes JM. Lung cancer in women. Lung Cancer Target Ther. 2012;3:79. 10.2147/LCTT.S37319 PMC5312492 28210127

[15] Olak J , Colson Y. Gender differences in lung cancer: have we really come a long way, baby? J Thorac Cardiovasc Surg. 2004;128:346-351. 15354089 10.1016/j.jtcvs.2004.05.025

[16] Medina FM , Barrera RR, Morales JF, et al Primary lung cancer in Mexico city: a report of 1019 cases. Lung Cancer. 1996;14:185-193. 8794402 10.1016/0169-5002(96)00545-4

[17] Pham D , Kris MG, Riely GJ, et al Use of cigarette-smoking history to estimate the likelihood of mutations in epidermal growth factor receptor gene exons 19 and 21 in lung adenocarcinomas. JCO. 2006;24:1700-1704. 10.1200/JCO.2005.04.3224 16505411

[18] Sharma SV , Bell DW, Settleman J, et al Epidermal growth factor receptor mutations in lung cancer. Nat Rev Cancer. 2007;7:169-181. 17318210 10.1038/nrc2088

[19] Shigematsu H , Gazdar AF. Somatic mutations of epidermal growth factor receptor signaling pathway in lung cancers. Int J Cancer. 2006;118:257-262. 16231326 10.1002/ijc.21496

[20] May L , Shows K, Nana-Sinkam P, et al Sex differences in lung cancer. Cancers (Basel). 2023;15:3111. 37370722 10.3390/cancers15123111 PMC10296433

[21] Stabellini N , Bruno DS, Dmukauskas M, et al Sex differences in lung cancer treatment and outcomes at a large hybrid academic-community practice. JTO Clin Res Rep. 2022;3:100307. 35400080 10.1016/j.jtocrr.2022.100307 PMC8983352

[22] Duque JL , Ramos G, Castrodeza J, et al Early complications in surgical treatment of lung cancer: a prospective, multicenter study. Ann Thorac Surg. 1997;63:944-950. 9124968 10.1016/s0003-4975(97)00051-9

[23] Chatkin JM , Abreu CM, Fritscher CC, et al Is there a gender difference in non-small cell lung cancer survival? Gender Med. 2004;1:41-47. 10.1016/s1550-8579(04)80009-3 16115582

[24] Visbal AL , Williams BA, Nichols FC, et al Gender differences in non–small-cell lung cancer survival: an analysis of 4,618 patients diagnosed between 1997 and 2002. Ann Thorac Surg. 2004;78:209-215; discussion 215. 15223430 10.1016/j.athoracsur.2003.11.021

[25] Ferguson MK , Wang J, Hoffman PC, et al Sex-associated differences in survival of patients undergoing resection for lung cancer. Ann Thorac Surg. 2000;69:245-249. 10654523 10.1016/s0003-4975(99)01078-4

[26] Strand T-E , Bartnes K, Rostad H. National trends in lung cancer surgery. Eur J Cardiothorac Surg. 2012;42:355-358. 22402451 10.1093/ejcts/ezs002

[27] Cerfolio RJ , Bryant AS, Scott E, et al Women with pathologic stage I, II, and III non-small cell lung cancer have better survival than men. Chest. 2006;130:1796-1802. 17166999 10.1378/chest.130.6.1796

[28] Scaglia NC , Chatkin JM, Pinto JA, et al Role of gender in the survival of surgical patients with nonsmall cell lung cancer. Ann Thorac Med. 2013;8:142-147. 23922608 10.4103/1817-1737.114297 PMC3731855

[29] Radkiewicz C , Dickman PW, Johansson ALV, et al Sex and survival in non-small cell lung cancer: a nationwide cohort study. PLoS One. 2019;14:e0219206. 31247015 10.1371/journal.pone.0219206 PMC6597110

[30] Gee K , Yendamuri S. Lung cancer in females—sex-based differences from males in epidemiology, biology, and outcomes: a narrative review. Transl Lung Cancer Res. 2024;13:163-178. 38405003 10.21037/tlcr-23-744 PMC10891406

[31] Langley JE , Sibley D, Chiekwe J, et al Prehabilitation program for lung and esophageal cancers (boosting recovery and activity through early wellness): protocol for a nonrandomized trial. JMIR Res Protoc. 2025;14:e60791. 40063931 10.2196/60791 PMC11933754

